# Testing for dependence on tree structures

**DOI:** 10.1073/pnas.1912957117

**Published:** 2020-04-22

**Authors:** Merle Behr, M. Azim Ansari, Axel Munk, Chris Holmes

**Affiliations:** ^a^Department of Statistics, University of California, Berkeley, CA 94720;; ^b^Department of Statistics, University of Oxford, Oxford OX1 3LB, United Kingdom;; ^c^Wellcome Centre for Human Genetics, University of Oxford, Oxford OX3 7BN, United Kingdom;; ^d^Institute for Mathematical Stochastics, University of Göttingen, Göttingen 37077, Germany;; ^e^Max Planck fellow group “Statistical Inverse Problems in Biophysics,” Max Planck Institute for Biophysical Chemistry, Göttingen 37077, Germany;; ^f^Cluster of Excellence “Multiscale Bioimaging: from Molecular Machines to Networks of Excitable Cells” (MBExC), University of Goettingen, Goettingen 37073, Germany;; ^g^The Alan Turing Institute, Health Data Research UK, London NW1 2BE, United Kingdom

**Keywords:** subgroup detection, hypothesis testing, tree structures, change-point detection

## Abstract

Tree structures, showing hierarchical relationships and the latent structures between samples, are ubiquitous in genomic and biomedical sciences. A common question in many studies is whether there is an association between a response variable measured on each sample and the latent group structure represented by some given tree. Currently, this is addressed on an ad hoc basis, usually requiring the user to decide on an appropriate number of clusters to prune out of the tree to be tested against the response variable. Here, we present a statistical method with statistical guarantees that tests for association between the response variable and a fixed tree structure across all levels of the tree hierarchy with high power while accounting for the overall false positive error rate. This enhances the robustness and reproducibility of such findings.

## Significance

Tree-like structures are abundant in the empirical sciences as they can summarize high-dimensional data and show latent structure among many samples in a single framework. Prominent examples include phylogenetic trees or hierarchical clustering derived from genetic data. Currently, users employ ad hoc methods to test for association between a given tree and a response variable, which reduces reproducibility and robustness. In this paper, we introduce treeSeg, a simple to use and widely applicable methodology with high power for testing between all levels of hierarchy for a given tree and the response while accounting for the overall false positive rate. Our method allows for precise uncertainty quantification and therefore, increases interpretability and reproducibility of such studies across many fields of science.

In the era of big data where quantifying the relationship between samples is difficult, tree structures are commonly used to summarize and visualize the relationship between samples and to capture latent structure. The hierarchical nature of trees allows the relationships between all samples to be viewed in a single framework, and this has led to their widespread usage in genomics and biomedical science. Examples are phylogenetic trees built from genetic data, hierarchical clustering based on distance measures of features of interest (for example, gene expression data with thousands of markers measured in each sample), evolution of human languages, and more broadly, in machine learning where clustering and unsupervised learning are fundamental tasks (1–7).

Often, samples have additional response measurements $y_{i}$ (e.g., phenotypes), and a common question is whether there is a relation between the sample’s latent group structure captured by the tree T and the outcome of interest $y_{i}$ (i.e., whether the distribution of $y_{i}$ depends on its relative location among the leaves of the tree T). Testing for all possible combinations of groupings on the tree is practically impossible as it grows exponentially with sample size. Currently, users typically decide on the number of clusters on an ad hoc basis (e.g., after plotting the response measurement on the leaves of the tree and deciding visually which clusters to choose), which are then tested for association with the outcome of interest. This lack of rigorous statistical methodology has limited the translational application and reproducibility of these methods.

Here, we present a statistical method and accompanying R package, treeSeg, that, given a significance level α, test for dependence of the response measurement distribution on all levels of hierarchy in a given tree while accounting for multiple testing. It returns the most likely segmentation of the tree such that each segment has a distinct response distribution while controlling the overall false positive error rate. This is achieved by embedding the tree segmentation problem into a change-point detection setting (8–13).

treeSeg does not require any assumptions on the generation process of the tree T. It treats T as given and fixed, testing the response of interest against the given tree structure. Every tree T, independent of how it was generated, induces some latent ordering of the samples. treeSeg tests whether, for this particular ordering, the distribution of the independent observations $y_{i}$ depends on their locations on the tree.

treeSeg is applicable to a wide range of problems across many scientific disciplines, such as phylogenetic studies, molecular epidemiology, metagenomics, gene expression studies, etc. (3, 5, 14–16), where the association between a tree structure and a response variable is under investigation. The only inputs needed are the tree structure T and the outcome of interest $y_{i}$ for the leaves of the tree. We demonstrate the sensitivity and specificity of treeSeg using simulated data and its application to a cancer gene expression study (1).

## Results

For ease of presentation, we restrict to discrete binary response measurements $y_{i}\in \left\{0,1\right\}$. However, the procedure is equally applicable to continuous and other observation types (*Methods*). If there is no association between the tree T and the response measurement $y_{i}$, then the observed responses $y_{i}$ would be randomly distributed on the leaves of the tree, independent of the tree structure T. However, if the distribution of responses is associated with the tree structure, we may observe clades in the tree with distinct response distributions. The power to detect segments with distinct distributions depends on the size of the clade and the change in response probability, $p_{i}=\text{P}\left(Y_{i}=1\right)=1-\text{P}\left(Y_{i}=0\right)$, which means that one can only make statistical statements on the minimum number of clades with distinct distributions on the tree and not the maximum.

**Fig. 1.**
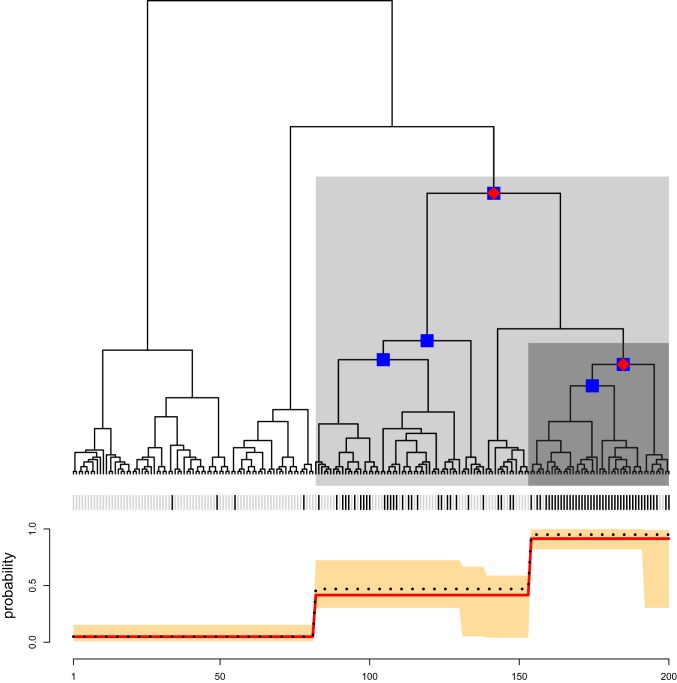
Illustration of the treeSeg method. Binary tree with 200 leaves and three segments with distinct response distributions indicated by dark gray, light gray, and white backgrounds. Outcomes for each sample are shown on the leaves of the tree as gray or black vertical lines. Leaf responses were simulated such that the black line has probabilities of 0.95,0.47, and 0.05 for each of the dark gray, light gray, and white background sections, respectively. Using α=0.1, treeSeg has estimated three segments on the tree with distinct response distributions indicated by the red diamonds on the nodes of the tree. Blue squares constitute a 90% confidence set for the nodes of the tree associated with the change in response distribution. *Lower* shows the simulation response probabilities (black dotted line), the treeSeg estimate (red line), and its 90% confidence bands (orange).

Fig. 1 illustrates our method and its output for a simulated dataset. The responses $y_{i}$ are displayed on the leaves of the tree as black and gray lines in Fig. 1. The tree T is made of three segments with distinct distributions over the responses indicated by dark gray, light gray, and white backgrounds in Fig. 1. Given a confidence level 1−α (e.g., 1−α=0.9,0.95), the treeSeg procedure estimates the most likely segmentation of the tree into regions of common response distributions such that the true number of segments is at least as high as the estimated number of segments with probability of 1−α.

Our method employs many likelihood ratio (LR) statistics simultaneously to test for changes in the response distribution on all levels of tree hierarchy and estimates at what level, if any, there is a change. The multiple testing procedure of treeSeg is based on a multiscale change-point methodology (11) tailored to the tree structure. The significance levels of the individual tests are chosen in such a way that the overall significance level is the prespecified α. As well as the maximum likelihood estimate, our method also provides confidence sets (at the 1−α level) for the nodes of the tree associated with the change in response distribution and a confidence band for the response probabilities $p_{i}$ over the segments (*Methods* and *SI Appendix* have theoretical proofs). In the example of Fig. 1, using α=0.1, treeSeg estimates three segments in the tree T, indicated with Fig. 1, red diamonds on the nodes of the tree, recovering the true simulated changes in response distributions. In Fig. 1, blue squares on the tree indicate the 1−α confidence set for the nodes on the tree associated with the change in responses $y_{i}$. The red line in Fig. 1, *Lower* shows the maximum likelihood estimate of the response probabilities $p_{i}$ for each segment, which accurately recovers the true simulated probabilities shown as the black dotted line. The orange band in Fig. 1, *Lower* shows the 1−α confidence band of the response probabilities.

The treeSeg method can handle missing data and make response predictions on new samples. Computationally, treeSeg scales well with sample size: for example, a test simulation for a tree with 100,000 samples (number of leaves in the tree) and no response association took around 110 min to run on a standard laptop. Details on treeSeg’s implementation are in *SI Appendix*.

### Simulation Study.

We confirmed the statistical calibration and robustness of treeSeg using simulation studies. We found that, for reasonable minimal clade sizes and changes in response distribution, treeSeg is able to detect association between response and tree structure reliably (*SI Appendix*, Figs. S11–S16). More importantly, treeSeg almost never detects segments that are not present (it can be mathematically proven that the inclusion of a false positive segment only happens with probability ≤α) (*Methods*), and the nominal guarantee of 1−α is exceeded in most cases. For instance, in a simulation study of 1,000 randomly generated trees (200 leaves) with no changes in response distribution, treeSeg (using α=0.1) correctly detected no association in 98.6% of the runs.

The treeSeg algorithm uses a fixed ordering of the leaf nodes according to the tree structure T. In principle, any such ordering is equally valid as long as this is made independent of the response variables. In our implementation in *SI Appendix*, we apply a standardized ordering of the nodes so that treeSeg’s output is independent of any user-specified ordering. Furthermore, we provide simulation results showing that treeSeg is robust to random changes in node ordering and consistently infers the correct number of segments on the tree (*SI Appendix*, section 2.B and Figs. S17–S25). In *SI Appendix*, section 2.B.1, we provide some discussion on signal-dependent branch orderings that may yield a higher detection power compared with others. We also present a procedure for aggregating results across random orderings to ensure that the output is independent of any specific tip ordering (*SI Appendix*, section 1.E). *SI Appendix* has full details on simulation studies.

The treeSeg procedure is conditioned on the input tree and thus, independent of the particular way that the tree is generated. In real applications, the input tree structure T is usually just a noisy version of some true neighborhood structure $\tilde{T}$ of interest. Therefore, whenever the noise in the input tree T is reasonably small such that T and $\tilde{T}$ essentially describe the same neighborhood structure, treeSeg’s output is robust to this noise (*SI Appendix*, section 1.F has further illustration).

### Application to Cancer Data Example.

We illustrate the application of the method on a breast cancer gene expression study (1) where data are publicly available. Following the original study, we used correlation of gene expression data as a distance measure between samples to build a hierarchical clustering tree. In the original study, based on visual inspection, the authors divided the samples into two clusters, observing differences in the distributions of various clinical responses between the two clusters.

**Fig. 2.**
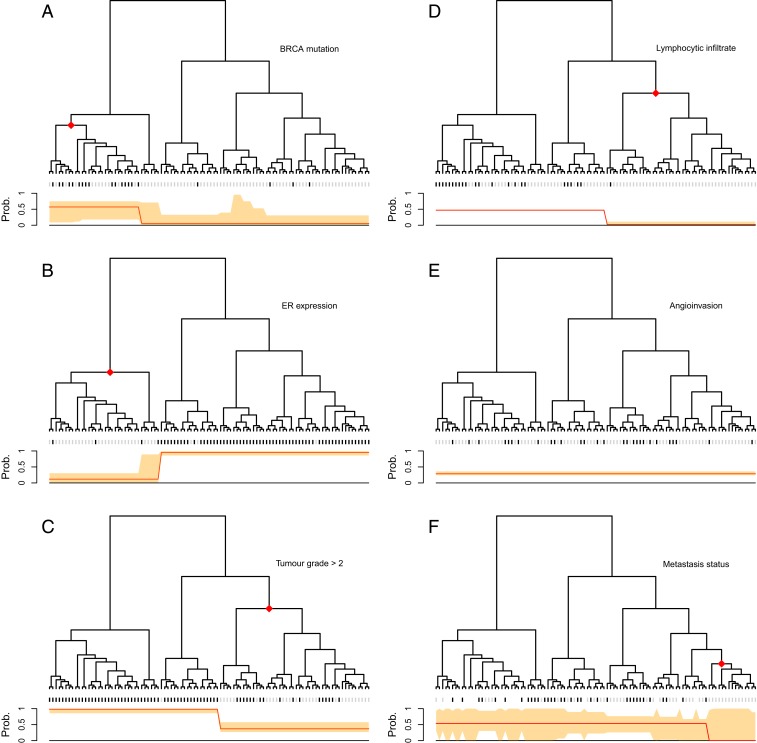
Application of treeSeg to a cancer gene expression study (1). Gene expressions for 98 breast cancer samples were clustered based on correlation between samples. Six clinical responses were collected for the samples (*A*) BRCA mutation, (*B*) estrogen receptor (ER) expression, (*C*) histological grade, (*D*) lymphocytic infiltration, (*E*) angioinvasion, and (*F*) development of distant metastasis within 5 y. In each panel, the treeSeg estimation (at α=0.05) for clades with distinct response distribution and their probabilities are indicated by the red diamonds on the tree and the red lines below the tree, respectively. The orange band shows the 95% confidence band for the response probabilities $p_{i}$ for the estimated segments. In *E*, there is no association between the tree and the response (angioinvasion), and in *F*, some of the samples have missing observations for the response (distant metastasis within 5 y).

In contrast, treeSeg only requires a significance level α as input and searches for associations between responses and the tree on all levels of hierarchy while accounting for multiple testing. Our results are shown in Fig. 2. Using an α=0.05, for one of the responses treeSeg delineated the tree into two clusters with distinct response distribution as in the original study. However, treeSeg reports different patterns of association between the tree and the other five responses, including one that has no association with tree structure.

The treeSeg algorithm can be applied to any tree structure and is not restricted to trees generated using hierarchical clustering. An application to a maximum likelihood phylogenetic tree generated from pathogen sequence data is in *SI Appendix*, section 3.

## Discussion

The only tuning parameter for the treeSeg method is a significance level α. Depending on the application, the user can decide which value of α is appropriate or screen through several values of α (e.g., α=0.01,0.05,0.1,0.5). A small α gives a higher confidence that all detected associations are, indeed, present in the data (with probability of at least 1−α). A larger α allows us to detect more clusters but increases the risk of including false positive clusters.

The confidence statement for detected clades and response probabilities that accompany treeSeg’s segmentation account for multiple testing at the level of 1−α. This allows for precise uncertainty quantification when detecting associations between tree structure and the responses. We highlighted treeSeg’s potential with an example from a gene expression study but note its ubiquitous applicability in various settings and its potential to be used across many fields of science. Our method treeSeg is implemented as an R package available on GitHub (https://github.com/merlebehr/treeSeg) and accompanied by a detailed Jupyter notebook with reproductions of all figures in this text.

## Methods

### Model Assumptions.

For illustration purposes, we focus on binary traits $Y_{i}\in \left\{0,1\right\}$. *SI Appendix*, section 1.B shows how treeSeg generalizes for arbitrary continuous and discrete data. We assume a fixed given rooted tree T with n leaves that captures some neighborhood structure of interest. For the n samples (the leaves of the tree), independent binary traits $Y_{i}$, i=1,…,n, with success probability $p_{i}$, are observed: that is,

$$Y_{i}∼\text{Bern}\left(p_{i}\right)\;\Leftrightarrow \;\text{P}\left(Y_{i}=1\right)=1-\text{P}\left(Y_{i}=0\right)=p_{i},$$ [1]

independently for i=1,…,n, where Bern(p) denotes a Bernoulli distribution with success probability p. The aim is to estimate the underlying success probabilities $p_{1},\ldots ,p_{n}$ from the observations $Y_{i}$. Without any additional structural information on the success probabilities, we cannot do better than estimating $p_{i}=Y_{i}$. However, taking into account the tree structure, we can assume that the success probabilities are associated with the tree such that samples on the same clade of the tree may have the same success probabilities. Our methodology is based on a testing problem, where the null model assumes that all isolates have the same success probability, say $p_{0}$, and the alternative model assumes that some of the clades on the tree have different success probabilities ($p_{0}+c\in \left[0,1\right]$). In the following, we denote an internal node (which demarcates a clade on the tree) with a distinct success probability as an active node.

For simplicity, we will assume in the following that the tree T is binary. Extensions to arbitrary trees are straight forward. We use the following notation. For a binary, rooted tree T=(V,E), we assume vertices V={1,…,n,n+1,…,2n−1} and edges E={(i,j) : i,j∈Vwithi,jconnected}. The leaves are labeled $V_{L}=\left\{1,\ldots ,n\right\}$, the inner nodes are labeled $V_{I}=\left\{n+1,\ldots ,2n-1\right\}$, and the root is labeled 2n−1. For a node i∈V, its set of offspring leaves in $V_{L}$ is denoted as Off(i). For a node i∈V, the subtree of T with root i is denoted as T(i). An illustrative example for this notation is shown in *SI Appendix*, Fig. S28. Moreover, for an inner node $i\in V_{I}$ with offspring leaves $\text{Off}\left(i\right)=\left\{i_{1},\ldots ,i_{m}\right\}\subset \left\{1,\ldots ,n\right\}$ and for some ϵ∈(0,1), we denote the left ϵ-leaf neighborhood of i as ${\text{N}}_{\text{L}}\left(i,\epsilon \right)=\left\{i_{1}-\left\lfloor n\epsilon \right\rfloor ,i_{1}-\left\lfloor n\epsilon \right\rfloor +1,\ldots ,i_{1}+\left\lfloor n\epsilon \right\rfloor -1,i_{1}+\left\lfloor n\epsilon \right\rfloor \right\}$ and analog, the right ϵ-leaf neighborhood of i as ${\text{N}}_{\text{R}}\left(i,\epsilon \right)=\left\{i_{m}-\left\lfloor n\epsilon \right\rfloor ,i_{m}-\left\lfloor n\epsilon \right\rfloor +1,\ldots ,i_{m}+\left\lfloor n\epsilon \right\rfloor -1,i_{m}+\left\lfloor n\epsilon \right\rfloor \right\}$.

We consider the following statistical model.

#### Model 1.

For a given binary, rooted tree T=(V,E) as above, assume that one observes for each of the leaves $i\in V_{L}$ independent Bernoulli random variables $Y_{i}∼B\left(p_{i}\right)$, 1≤i≤n, where the vector of success probabilities $p=\left(p_{1},\ldots ,p_{n}\right)$ is an element of $S=S\left(T\right)≔\left\{{\left(p_{0}+\sum_{j=1}^{k}c_{j}1_{i\in \text{Off}\left(v_{j}\right)}\right)}_{1\leq i\leq n}\in {\left[0,1\right]}^{n}:\;v_{j}\in V,\;p_{0},c_{j}\in R,\;0\leq k\leq 2n-1\right\}$.

For an element p∈S, we denote the set of nodes $V\left(p\right)≔\left\{v_{1},\ldots ,v_{k}\right\}$ as a set of active nodes and k(p)=k as the number of active nodes. To ensure identifiability of active nodes, we further assume that, for each active node $v_{j}$, j=1,…,k, there exists at least one offspring leaf i∈1,…,n that has the same success probability as $v_{j}$. This just excludes the trivial case where the influence of one active node (or the root) is completely masked by other active nodes. Equivalently, this means that we assume that $\#\text{supp}\left(p\right)=\#\left\{p_{i}\;:\;i=1,\ldots ,n\right\}=k+1$. We provide a simple example in *SI Appendix*, Fig. S30.

We stress that the set V(p) is not necessarily unique (*SI Appendix*, Fig. S29 has an example). That is, for a given vector p∈S, there may exist two (or more) sets of active nodes $\left\{v_{1},\ldots ,v_{k}\right\}$ and $\left\{v_{1}^{\prime },\ldots ,v_{k}^{\prime }\right\}$ such that $p_{i}=p_{0}+\sum_{j=1}^{k}c_{j}1_{i\in \text{Off}\left(v_{j}\right)}=p_{0}^{\prime }+\sum_{j=1}^{k}c_{j}^{\prime }1_{i\in \text{Off}\left(n_{j}^{\prime }\right)}$. To overcome this ambiguity, we will implicitly associate with each p∈S(T) a set of active nodes V(p) of size k(p). When a specific vector p∈S(T) has more than one possible sets of active nodes $V^{\prime },V^{\prime \prime },\ldots$, we assume several copies of p in S(T), one associated with each of the sets $V^{\prime },V^{\prime \prime },\ldots$. In the following, we explore the tree structure to estimate the underlying success probabilities $p_{i}$ and hence, their segmentation into groups of leaves where observations within a group have the same success probability and observations between different groups have different success probabilities.

### Multiscale Segmentation.

The procedure that we propose extends (11) from totally ordered structures to trees and is a hybrid method of estimating and testing. A fundamental observation is that one can never rule out an additional active node. This is because a node could be active but change the success probability of its offspring nodes only by an arbitrarily small amount. On the other hand, if in a subtree, successes are much more common than in the remaining tree, it is possible to significantly reject the hypothesis that all leaves in the tree have the same success probability.

For a given candidate vector $\tilde{p}\in S$, our procedure employs on each subtree T(i) where $\tilde{p}$ is constant, with $\tilde{p}\equiv \tilde{p}\left(\text{Off}\left(i\right)\right)$, an LR test for the hypothesis that the corresponding observations all have the same success probability $\tilde{p}\left(\text{Off}\left(i\right)\right)$. The levels of the individual tests are chosen in such a way that the overall level of the multiple test is α for a given prespecified α∈(0,1). A statistical hypothesis test can always be inverted into a confidence statement and vice versa. Therefore, we can derive from the above procedure a confidence set for the vector of success probabilities $p=\left(p_{1},\ldots ,p_{n}\right)$. We require our final estimate $\hat{p}={\hat{p}}_{1-\alpha }$ to lie in this confidence set. That is, we require that, whenever $\hat{p}$ has constant success probability on a subtree, the respective LR test accepts. Within all vectors p∈S that lie in this confidence set, we choose one which comes from a minimum number of active nodes, and within this set, we choose the maximum likelihood solution. Thereby, our procedure not only provides an estimate but also, provides a confidence statement for all quantities (11). More precisely, the following asymptotic confidence statements hold true.

1) With probability at least 1−α, the true underlying signal p∈S originates from at least $\hat{k}$ active nodes, where $\hat{k}$ is the number of active nodes of $\hat{p}$ (Theorem 1).
2) treeSeg yields a set of nodes $C_{1-\alpha }$, such that the active nodes of p, V(p), are contained with probability at least 1−α in $C_{1-\alpha }$ (Corollary).
3) treeSeg yields a confidence band for the underlying signal p, denoted as ${\underline{p}}_{1-\alpha }$ and ${\bar{p}}_{1-\alpha }$, such that with probability at least 1−α it holds that ${\underline{p}}_{1-\alpha }\leq p\leq {\bar{p}}_{1-\alpha }$ simultaneously for all i=1,…,n (Theorem 3).

Moreover, the coverage probability of the confidence sets allows us to derive (up to log factors) optimal convergence rates of the treeSeg estimator as the sample size n increases (11). In particular, we show the following.

4) For fixed overestimation bound α∈(0,1), the probability that treeSeg underestimates the number of active nodes k vanishes exponentially as n increases (Theorem 2).
5) The localization of the estimated active nodes is optimal up to a leaf node set of order log(n) (Theorem 4).

In the following, we will give details of the method and of the statements 1 to 5. The proofs of Theorems 1 to 4 and Corollary are similar to the totally structured setting (11). Necessary modifications are outlined in *SI Appendix*.

For an arbitrary given test vector $\tilde{p}$ (which may depend on Y), we define the multiscale statistic (11, 17–19)

$$T_{n}\left(\tilde{p},Y\right)=\underset{\begin{array}{c} 1\;\leq \;i\;\leq \;j\;\leq \;n\\ \tilde{p}{|}_{\left[i,j\right]}\text{const.} \end{array}}{\max}\sqrt{2T_{i}^{j}\left(Y_{i}^{j},{\tilde{p}}_{i}\right)}-pen\left(\;j-i+1\right),$$ [2]

where $Y_{i}^{j}=\left(Y_{i},\ldots ,Y_{j}\right)$ and $pen\left(x\right)≔\sqrt{2\log\left(e/x\right)}$. Here, $T_{i}^{j}$ is the local log LR test statistic (20) for the testing problem

$$H:\;p_{i}=\ldots =p_{j}=\tilde{p}{|}_{\left[i,\;j\right]}\;\text{vs.}\;K:\;p_{i}=\ldots =p_{j}\neq \tilde{p}{|}_{\left[i,\;j\right]}.$$

The calibration term pen(⋅) serves as a balancing of the different scales in a way that the maximum in Eq. **2** is equally likely attained on all scales (11, 17) and guarantees certain optimality properties of the statistic [**2**] (17). Assuming a minimal segment scale λ∈(0,1) of the underlying success probability vector p: that is,

$$S_{\lambda }=\left\{p\in S\text{with const. segments’ length at least}\;n\lambda \right\},$$ [3]

it can be shown that $T_{n}\left(p,Y\right)$ converges in distribution to a functional of the Brownian motion (11), which is stochastically bounded by

$$M≔\underset{0\leq s<t\leq 1}{\sup}\left(\frac{B\left(t\right)-B\left(s\right)}{\sqrt{t-s}}-\sqrt{2\log\frac{e}{t-s}}\right).$$ [4]

Thereby, the minimal scale λ may depend on n such that $n\lambda /log{\left(n\right)}^{3}\to \infty$ as n→∞ (11). As the distribution of M does not depend on the true underlying signal p, its quantiles can be obtained by Monte Carlo simulations and are in the following denoted as $q_{1-\alpha }$: that is,

$$\underset{n\to \infty }{\lim}\underset{p\in S_{\lambda }}{\sup}\text{P}\left(T_{n}\left(p,Y\right)>q_{1-\alpha }\right)\leq \text{P}\left(M>q_{1-\alpha }\right)=\alpha .$$ [5]

For a given confidence level α∈(0,1) or equivalently, a threshold value $q=q_{1-\alpha }$ in Eq. **5**, we first define an estimator for the number of active nodes k in Model 1 via

$$\hat{k}\left(q\right)≔\underset{p\in S}{\min}k\left(p\right)\;\text{s.t.}T_{n}\left(p,Y\right)\leq q.$$ [6]

After the number of active nodes k is estimated, we estimate p as the constrained maximum likelihood estimator

$$\hat{p}\left(q\right)≔{argmax}_{p\in H\left(q\right)}\overset{n}{\underset{i=1}{\sum }}l_{p_{i}}\left(Y_{i}\right),$$ [7]

where $l_{p}\left(y\right)$ is the log likelihood function of the binomial distribution and

$$H\left(q\right)≔\left\{p\in S:k\left(p\right)=\hat{k}\left(q\right)\text{and}T_{n}\left(p,Y\right)\leq q\right\}.$$ [8]

Note that the maximum likelihood solution in Eq. **7** is not necessarily unique. On the one hand, this is due to the nonuniqueness of the active nodes. On the other hand, this might happen with positive probability by the discreteness of the Bernoulli observations Y. In that case, treeSeg just reports the first available solution, with all other equivalent solutions listed in the confidence set. Clearly, if we choose q as in Eq. **5** for some given confidence level α∈(0,1), the estimator $\hat{k}\left(q_{1-\alpha }\right)$ asymptotically controls the probability to overestimate the number of active nodes as summarized in the following theorem.

#### Theorem 1.

*For fixed minimal scale* λ>0 *and significance level* 1−α∈(0,1), *let* $S_{\lambda }$ *be as in* *Eq.* **3**, $q_{1-\alpha }$ *be as in* *Eq.* **5**, *and* $\hat{k}\left(q\right)$ *be treeSeg*’*s estimated number of active nodes in* *Eq.* **6**. *Then, it holds that*

$$\underset{n\to \infty }{\lim}\underset{p\in S_{\lambda }}{\sup}\text{P}\left(\hat{k}\left(q_{1-\alpha }\right)>k\left(p\right)\right)\leq \alpha .$$ [9]

We stress that, in Theorem 1, it is possible to let λ go to zero as n increases (11); recall the paragraph after Eq. **4**. In particular, from the construction of $\hat{k}$ in Eq. **6**, it follows that $T_{n}\left(p,Y\right)\leq q$ implies $\hat{k}\left(q\right)\leq k$, and thus, for the set $H\left(q_{1-\alpha }\right)$ in Eq. **8**, one obtains that

$$\text{P}\left(p\in H\;\left(q_{1-\alpha }\right)\right)\geq \text{P}\left(T_{n}\left(p,Y\right)\leq q_{1-\alpha }\right)-\text{P}\left(\hat{k}\;\left(q_{1-\alpha }\right)<k\right).$$ [10]

By Eq. **5**, it follows that the first term on the right-hand side, $\text{P}\left(T_{n}\left(p,Y\right)\leq q_{1-\alpha }\right)$ , is asymptotically lower bounded by 1−α. Moreover, as we show in Theorem 2, the underestimation error $\text{P}\left(\hat{k}\;\left(q_{1-\alpha }\right)<k\right)$ vanishes exponentially fast as sample size n increases. From this, it follows that the set $H\left(q_{1-\alpha }\right)$ constituents an asymptotically honest confidence set (11) for the whole vector p from which confidence sets for the active nodes and confidence bands for p as in statements 2 and 3 follow (Theorem 3 and Corollary).

Any bound on the underestimation error necessarily must depend on the minimal segment scale λ in Eq. **3** as well as a minimal pairwise difference δ∈(0,1) of success probabilities in different active segments. That is, for $p\in S_{\delta }$, we assume $\delta <\underset{p_{i}\neq p_{j}}{\min}\left|p_{i}-p_{j}\right|$ and let

$$S_{\delta ,\lambda }=S_{\lambda }\cap S_{\delta }.$$ [11]

With this, one obtains that the underestimation probability decreases exponentially (11) in n [for fixed δ,λ and significance level 1−α∈(0,1)] as the following theorem shows.

#### Theorem 2.

*For fixed minimal scale* λ>0, *minimal probability difference* δ>0, *and significance level* 1−α∈(0,1), *let* $S_{\lambda ,\delta }$ *be as in* *Eq.* **11**, $q_{1-\alpha }$ *be as in* *Eq.* **5**, *and* $\hat{k}\left(q\right)$ *be treeSeg*’*s estimated number of active nodes in* *Eq.* **6**. *Then*, *it holds that*

$$\underset{p\in S_{\lambda ,\delta }}{\sup}\text{P}\left(\hat{k}\left(q_{1-\alpha }\right)<k\left(p\right)\right)\leq C_{1}\;e^{-C_{2}\;n},$$ [12]

*where* $C_{1}$ *and* $C_{2}$ *are positive constants*, *which only depend on* α,λ,δ.

Again, it is possible to let α,λ, and δ go to zero as the sample size n increases (11). The proof of Theorem 2 is similar as for totally ordered structures (11). We outline necessary modifications in *SI Appendix*. From Theorem 2 and [**10**], we directly obtain that $H\left(q_{1-\alpha }\right)$, indeed, constitutes a 1−α asymptotic confidence set for the segmentation p.

#### Theorem 3.

*For fixed minimal scale* λ>0, *minimal probability difference* δ>0, *and significance level* 1−α∈(0,1), *let* $S_{\lambda ,\delta }$ *be as in* *Eq.* **11**, $q_{1-\alpha }$ *be as in* *Eq.* **5**, *and* $H\left(q_{1-\alpha }\right)$ *be as in* *Eq.* **8**; *then*,

$$\underset{n\to \infty }{\lim}\underset{p\in S_{\lambda ,\delta }}{\sup}\text{P}\left(p\in H\left(q_{1-\alpha }\right)\right)\geq 1-\alpha .$$

*As a corollary*, *we also obtain a confidence set of the active nodes*.

#### Corollary.

*For fixed minimal scale* λ>0, *minimal probability difference* δ>0, *and significance level* 1−α∈(0,1), *let* $S_{\lambda ,\delta }$ *be as in* *Eq.* **11**, $q_{1-\alpha }$ *be as in* *Eq*. **5**, *and* $H\left(q_{1-\alpha }\right)$ *be as in* *Eq*. **8**; *then*,

$$\underset{n\to \infty }{\lim}\underset{p\in S_{\lambda ,\delta }}{\sup}\text{P}\left(V\left(p\right)\subset \left\{v\in V\left(\tilde{p}\right)\;:\;\tilde{p}\in H\left(q_{1-\alpha }\right)\right\}\right)\geq 1-\alpha .$$

Theorems 1 and 2 reveal treeSeg’s ability to accurately estimate the number of active nodes in Model 1. For any (arbitrarily small) α∈(0,1), we can control the overestimation probability by 1−α (Theorem 1). Simultaneously, as the sample size n increases, the underestimation error probability vanishes exponentially fast (Theorem 2). The next theorem shows that treeSeg does not just estimate the number of active nodes correctly with high probability but that it also estimates the location of those active nodes with high accuracy. To this end, note that, for any active node v∈V(p) and any ϵ≥1/n, the leaf nodes of its left ϵ-leaf neighborhood ${\text{N}}_{\text{L}}\left(v,\epsilon \right)$ have nonconstant success probability. The same is true for the right ϵ-leaf neighborhood ${\text{N}}_{\text{R}}\left(v,\epsilon \right)$. Now assume that treeSeg estimates the number of active nodes correctly $\hat{k}=k$, which is the case with high probability by Theorems 1 and 2. Then, $\hat{p}$ being nonconstant on both ${\text{N}}_{\text{R}}\left(v,1/n\right)$ and ${\text{N}}_{\text{L}}\left(v,1/n\right)$ for any true active nodes v∈V(p), implies a perfect segmentation. Thus, the following theorem shows that treeSeg, indeed, yields such a perfect segmentation up to a leaf node set of size O(log(n)). That is, conditioned on the correct model dimension $\hat{k}=k$, treeSeg’s segmentation is perfect up to at most an order of log(n) misclassified leaf nodes.

#### Theorem 4.

*For fixed minimal scale* λ>0, *minimal probability difference* δ>0, *and significance level* 1−α∈(0,1), *for any* $p\in S_{\lambda ,\delta }$ *it holds true that*

$$\hat{p}{|}_{{\text{N}}_{\text{L}}\left(v,\frac{C_{3}\log\left(n\right)}{n}\right)}\text{and}\hat{p}{|}_{{\text{N}}_{\text{R}}\left(v,\frac{C_{3}\log\left(n\right)}{n}\right)}$$

*are not constant*, *for all* v∈V(p), *with probability at least* $1-C_{1}\;e^{-C_{2}\;n}$, *where* $C_{1},C_{2},C_{3}$ *are positive constants that only depend on* α,λ,δ.

Theorem 4 follows directly by translating change-point location estimation accuracy results for the totally ordered case (11) to the tree setting.

A natural question is whether the localization rate in Theorem 4 is optimal. In particular, one can compare this result with the totally ordered setting, where the minimax optimal change-point estimation rate is known to be of the same order [possibly up to log(n) factors]. One would expect that the additional tree structure leads to a strictly better segmentation rate. It turns out, however, that without making further assumptions on the tree the rate in Theorem 4 cannot be improved in general (*SI Appendix*, Theorem 6). More precisely, for an arbitrary number of observations n, there always exist trees that do not contain any additional information other than the ordering of the tips. In that case, the tree structured setting is essentially equivalent to a regular change-point setting, and thus, treeSeg cannot yield any better performance.

On the other hand, when one imposes additional structural assumptions on the tree, it can be shown that treeSeg yields a perfect segmentation with high probability *SI Appendix*, Theorem 7. Essentially, when a tree structure is such that the segmentation from different sets of actives nodes is either the same or differs by some nonvanishing fraction γ∈(0,1) (more precisely, this is captured by the γ-spreading property in *SI Appendix*, Definition 1), then treeSeg will recover those active nodes exactly with high probability. A simple example of trees that provide such additional structure are perfect trees, where all tip nodes have the same depth. In summary, treeSeg efficiently leverages the tree structure to overcome the minimax lower bound from a simple change-point estimation problem whenever the tree allows this. We provide more details in *SI Appendix*, section 1.D.

## Supplementary Material

Supplementary File
